# Optimal Distributed MQTT Broker and Services Placement for SDN-Edge Based Smart City Architecture

**DOI:** 10.3390/s22093431

**Published:** 2022-04-30

**Authors:** Dzaky Zakiyal Fawwaz, Sang-Hwa Chung, Chang-Woo Ahn, Won-Suk Kim

**Affiliations:** Department of Computer Engineering, Pusan National University, Busan 46241, Korea; dzakyzf@pusan.ac.kr (D.Z.F.); changwooahn@pusan.ac.kr (C.-W.A.); wonsukkim@pusan.ac.kr (W.-S.K.)

**Keywords:** internet of things (IoT), software-defined networking, edge computing, distributed MQTT, container placement, smart city

## Abstract

A smart city is an urban area that collects data from various devices to effectively manage urban resources. The smart city IoT infrastructure connects numerous devices to an Internet-protocol-based low-power wireless network, shares massive amounts of data, and facilitates the development of new services. Message queuing telemetry transport (MQTT), a lightweight exchange protocol for the IoT environment, uses a publish and subscribe structure via a centralized broker to share data. The extent of edge computing provides distributed and closer resources to the data source while maintaining low transmission costs. However, a centralized MQTT data broker is unsuitable for distributed edge resources and could result in high latency, traffic, and bottleneck risk. Therefore, we proposed a distributed MQTT broker optimized architecture. A distributed MQTT broker for edge resources could reduce network traffic and data delivery latency by only managing consumed topics in the network. We formulate an integer non-linear program to optimize container placement and avoid wasting edge computing resources. We compared our proposed architecture to the existing distributed MQTT middleware architecture with greedy and random container placement through extensive simulation. Our methods show better performance in lowering deployment failure ratio, power consumption, network usage, and synchronization overhead.

## 1. Introduction

Many countries are currently working on projects to build smart cities using Internet of Things (IoT) technology [1]. Smart cities include smart energy, smart traffic, smart buildings, smart government, smart water management, and smart health care [2]. The number of connected devices to the Internet is expected to reach over 100 billion by 2025, including the smart city business [3]. The smart city IoT infrastructure connects a vast number of devices to an Internet-protocol-based low-power wireless network. The infrastructure needs to share a large amount of data generated by these devices and facilitate the creation of new services. Moreover, a widely used cloud computing model has faced numerous challenges. This is due to the data explosion caused by the proliferation of devices and the demand for low latency and real-time response. Edge computing has been proposed to overcome existing cloud computing limitations [4,5,6]. Edge computing extends existing cloud services such as computing, storage, and networks closer to devices or users. In other words, some cloud computing roles are distributed across switches, gateways, and mobile base stations. Edge computing is characterized by low latency, location awareness, and mobility support. A connected car system is an example of an application that can benefit from these features. It is critical to provide real-time driving conditions and directions to a fast-moving vehicle using nearby computing resources.

Meanwhile, software-defined networking (SDN) separates the control and data planes of network switches. An SDN controller sets forwarding rules to control the data plane flow. SDN enables the rapid adoption of new IoT protocols and policies. It can manage devices and networks based on statistics. A network computing resource could be provided by an edge-enabled SDN switch, which allows them to deploy container services. Edge computing can be used for crowdsensing [7]. Crowdsensing allows people with mobile devices to collect and share data to measure, analyze, and estimate common concerns. Applications of crowdsensing include noise pollution mapping, urban planning, and weather forecasting [8]. However, unexpected events may cause a data explosion. In this case, data can be processed locally using edge computing resources to reduce network traffic. IoT applications require a protocol with real-time response, low bandwidth, and low energy. The MQTT (message queuing telemetry transport) protocol is suitable for IoT. MQTT, a publish/subscribe messaging protocol for pervasive networks, can improve network bandwidth and device battery life [9]. MQTT organized data into topics and a publisher sends a message to the centralized broker to distribute data. The broker delivers data to topic subscribers and allows connecting many constrained devices and delivering data efficiently. MQTT has been used as a messaging protocol on IoT-based smart cities [10,11].

**Figure 1:**
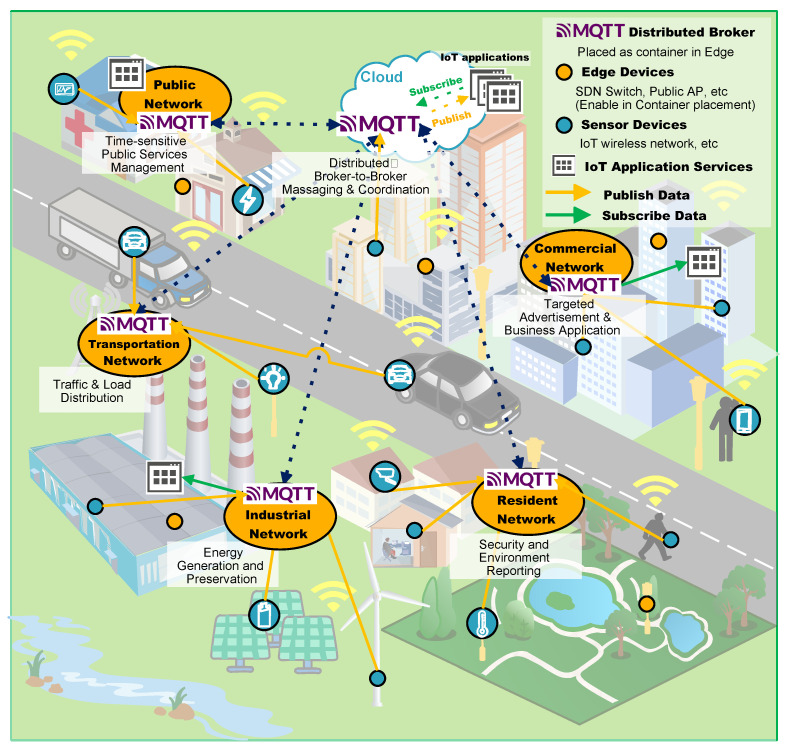
Distributed MQTT architecture based on edge computing in smart city application.

This paper aims to propose distributed MQTT broker architecture and service container placement optimization over distributed edge computing resources [12]. The distributed MQTT broker reduces latency and traffic load by operating close to edge resources. Because the edge resource is limited, we need to deploy only the required application services into the distributed resources. The edge device hosts the IoT service and distributed MQTT broker instances. The proposed architecture can support low latency and real-time smart city IoT services. The following items are considered when designing an edge-computing-based distributed MQTT architecture. If the MQTT broker function is implemented in the IoT service container, then the implementation complexity of the application service container increases. It is also difficult to clearly determine which services should have this functionality when multiple IoT services use the same topic. Removing a service container may also be impossible since it contains an MQTT broker that delivers MQTT data to another service container in an edge computing environment. As a result, edge computing resources are wasted. Therefore, the IoT service container and the distributed MQTT broker container should be set up and run separately to be easily managed. Next, the distributed broker must avoid wasting edge computing resources, thus not letting the IoT service be operated. Figure 1 depicts the above-mentioned distributed MQTT architecture. Based on these considerations, we propose a novel distributed MQTT broker architecture that only manages topics that are intensively produced or consumed locally.

The main contributions of this paper are as follows:

- We designed a distributed MQTT broker architecture based on edge computing and presented its detailed operation procedure;
- The proposed architecture facilitates the creation of edge-based smart city IoT services, which allows for close processing locally consumed data to mitigate data explosion;
- The proposed architecture efficiently utilizes edge computing resources by managing only the specific topics that are intensively consumed in the network or are used by the IoT service operated in the edge computing environment;
- To efficiently use limited edge computing resources, we propose the use of a container placement optimization scheme to minimize the power consumption of the edge computing resources.

The remainder of this paper is organized as follows. Section 2 presents related works, and Section 3 and Section 4 describe the edge-computing-based distributed MQTT architecture and introduce container placement optimization to minimize power consumption in the edge. Next, Section 5 and Section 6 analyze the simulation results and discuss the implications of the proposed method. Finally, Section 6 concludes the paper.

## 2. Related Work

In a smart-city infrastructure network, a server/client messaging model is expected to cause data explosion and high load concentration problems in the cloud. Hence, the publisher/subscriber model is gaining traction [13,14,15]. A broker relays messages between publishers and subscribers in this model. By registering a topic, subscribers are notified when new data on that topic is generated by publishers. MQTT is a popular protocol for this type of messaging. MQTT in smart cities has been studied extensively. Tantitharanukul et al. [2] proposed an efficient topic-naming criterion for sharing open data generated in smart cities. The data’s purpose, location, and owner were used as topic-naming criteria. Zabasta et al. [10] designed a smart city utility system to manage power, water, and the environment. An event-processing function is embedded in an MQTT broker in this system. Sultana et al. [16] developed an intelligent surveillance system based on image sensing and the Internet of Video Things. Among the application layer protocols tested, MQTT has the lowest protocol overhead and the highest throughput for sending images. Tsai et al. [17] used MQTT for vehicle networks and demonstrated the MQTT protocol’s ability to efficiently share low-power and high-bandwidth data. A distributed MQTT broker architecture can significantly reduce network traffic by managing such visual data locally.

The subsequent studies used MQTT in an edge computing environment. Xu et al. [18] implemented an SDN-based edge device prototype and installed an MQTT broker to analyze the message delivery performance. This paper showed that the distributed broker functionality could be run on edge devices. Rausch et al. [19] proposed EMMA, a distributed MQTT middleware architecture for edge computing. This architecture supports client mobility and dynamic broker provisioning via network reconfiguration. EMMA connects clients to distributed MQTT brokers based on load and distance, regardless of the topic. In this architecture, synchronization overhead between brokers can be greater depending on the topic used by clients connected to each distributed broker. Kawaguchi et al. [20] proposed a distributed broker system for large-scale location-based IoT services. Load balancing between brokers is handled automatically by a location-based hierarchical topic structure. Faticanti et al. [21] proposed an edge computing orchestration problem as a mixed-integer nonlinear optimization problem. It is then solved as an integer linear programming problem using a greedy algorithm. Unlike our work, this work did not consider a distributed broker. Various studies have been conducted to reduce power consumption by efficiently deploying virtual machines in a cloud environment [22,23]. These studies reduce the number of physical devices in which virtual machines are deployed through a bin packing algorithm in a cloud environment. Nonetheless, these methods are not suitable for edge computing. The edge computing environment can comprise networking devices such as routers and switches, which must maintain their operating state even if no virtual machine is deployed. Nishio et al. [24] proposed service-oriented utility functions and optimized service delay for heterogeneous resource sharing. Arkian et al. [25] proposed a hierarchical edge computing architecture for crowdsensing called MIST. The authors proposed using a container placement optimization method to reduce system maintenance costs. Mseddi et al. [26] proposed a meta-heuristic based on particle swarm optimization to solve the joint problem of service placement and task provisioning in fog/edge environments to maximize the number of served clients. Zhang et al. [27] study the container placement to optimize the energy consumption of virtual machines and propose an improved genetic algorithm (GA). Ibrar et al. [28] proposed the optimal position’s placement for fog nodes using singular value decomposition (SVD) and QR factorization on the network’s traffic matrix while also formulating routing optimization for job offloading using ant colony optimization (ACO). Moreover, meta-heuristics algorithms such as PSO, GA, and ACO have a risk of falling into a local optimal solution.

These previous approaches include several drawbacks. First, most of them were not considered an edge computing environment where the distributed broker is dynamically created/removed. Second, these studies do not consider how to efficiently deliver public data from smart cities within distributed edge computing resources. With the aforementioned issues, we propose distributed MQTT broker architecture and service container placement optimization over distributed edge computing resources. We formulated a container placement optimization problem into an integer non-linear program that aims to prevent the unnecessary use of edge computing resources. The significance of the present study lies in the development of novel distributed MQTT broker architecture that manages only specific topics that are intensively produced or consumed in the local network.

## 3. Distributed MQTT Broker Architecture Based on Edge Computing

### 3.1. Considerations for Designing Distributed MQTT Architecture

**Figure 2:**
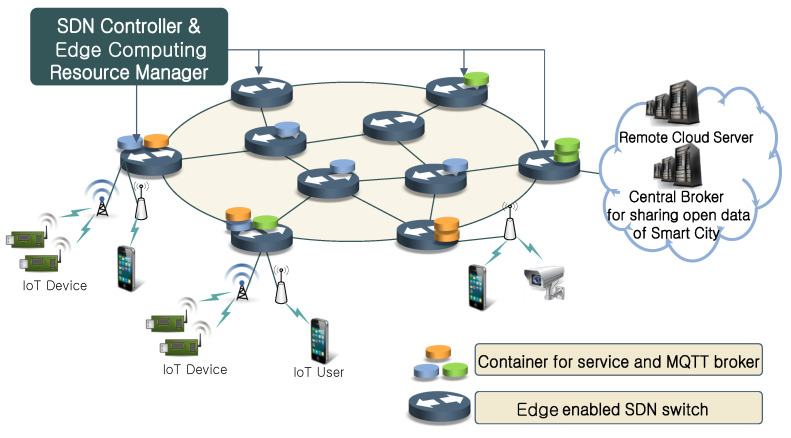
Edge computing environment.

Figure 2 shows the edge computing environment where the distributed MQTT architecture proposed in this paper is applied. The network consists of SDN switches with edge computing resources. The SDN switches can operate IoT services and distributed MQTT broker instances using Docker, a container-based virtualization tool. Each switch performs flow routing for the application service container placement/operation under the control of the SDN controller. The smart city infrastructure consists of networks owned by various owners. The central data broker for the smart city’s open data is hosted in a remote cloud, as are IoT application services. In this environment, the network owner can use edge computing resources to run an MQTT broker or IoT service application, reducing network traffic and improving QoS and user experience (UX). The cloud has high latency due to the long-distance transmission of large data. Edge computing addresses this issue by locating computational resources close to the data source, reducing latency and transmission costs. For example, edge devices could collect data from nearby IoT devices and directly process them with the deployed service container, eliminating unnecessary high latency and network bandwidth competition. Edge devices can either directly process data and send the insight to the cloud or preprocess and extract features from data and send it to the cloud with less complexity. As edge devices have limited resources, it is necessary to optimize service container allocation. We discussed and proposed a method to solve it in Section 4.

The smart-city IoT or cloud service providers can lease edge computing resources from the network owner to mitigate the server load and provide a fast response time of IoT services. Leasing edge computing resources to IoT and cloud service providers can be lucrative for network owners [6,29]. Local MQTT brokers in container form can be run on network devices with edge computing resources. It can reduce network traffic and data delivery latency in smart city environments with central MQTT brokers for open data sharing/delivery. Clients must be able to publish/subscribe to all topics regardless of the connected broker. It requires interoperation between the central broker and the local broker.

**Figure 3:**
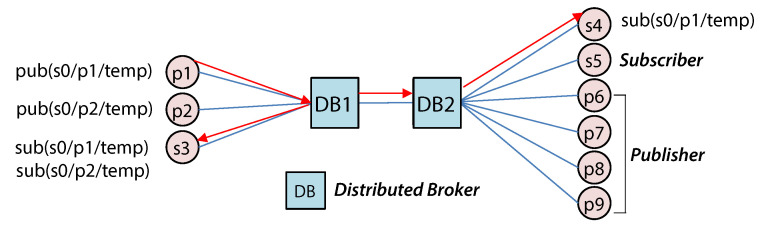
MQTT message delivery process between distributed brokers.

Figure 3 shows an operational example of an existing distributed broker architecture. MQTT clients (publishers/subscribers) are connected to DB1 and DB2, which are adjacent distribution brokers. When DB1 receives a message (e.g., s0/p1/temp) published by p1, it transmits the message to subscriber s3. It also transmits the message to DB2 to deliver to subscriber s4. DB1 needs to know that there is a subscriber to the topic published by p1 in DB2 in advance. Subscription flooding (SF) is used in the existing distributed broker architecture. The subscription message is sent to all distributed brokers to synchronize the subscription tables. Although there is a publication broadcasting method that broadcasts received publication messages to all distributed brokers regardless of the presence of subscribers, it is rarely used in the distributed broker architecture because of the flooding overhead of publication messages with a higher frequency than the subscription message. If the SF method is used on a distributed local MQTT broker in an edge computing environment, the distributed MQTT broker must keep a forwarding table for all MQTT topics. To work with the central broker, the local broker must forward all topic subscription messages to the central broker and maintain a subscription table for all central broker clients. Due to resource constraints, a single distributed MQTT broker container on an edge device cannot process all client subscription/publication messages. Synchronizing subscription tables between distributed broker containers also incurs additional overhead. If the computing resources required to operate a distributed broker exceed the network’s edge computing resources, the distributed broker cannot be deployed. This distributed broker architecture also wastes edge computing resources by processing/managing non-subscribed topic messages.

**Figure 4:**
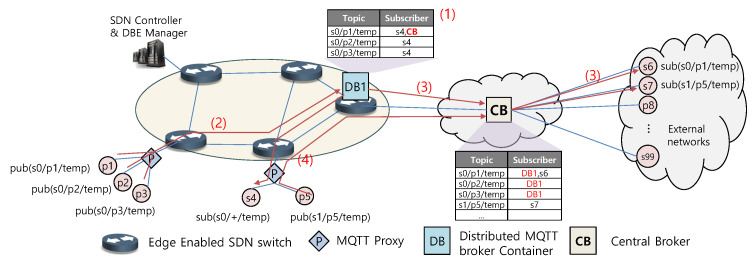
Operation of the distributed broker architecture of the proposed method.

As a result of the inefficient use of edge computing resources and the ability to operate across multiple edge devices in the network, we propose a novel distributed MQTT broker architecture that manages specific topics. The distributed broker manager delegates topics from a central broker to a distributed broker instance that manages the topic as a container in the edge device. With the proposed architecture, the central broker’s traffic is reduced and topic delivery latency is improved. A distributed MQTT broker container can be started or moved over edge devices. Since the MQTT protocol relies on TCP connections, the broker and client must first establish TCP connections before sending or receiving the MQTT protocol. The MQTT protocol then exchanges packets for the MQTT connection, publication, and subscription. The MQTT protocol does not provide a separate protocol for redirecting existing clients to other brokers. Even if the SDN controller changes the TCP flow forwarding rule, the TCP-based broker and client connection is lost. The dynamic creation, movement, and deletion of distributed brokers in the edge computing environment requires reestablishing client connections. Figure 4 demonstrates an operational example of the distributed MQTT architecture proposed in this paper. The proposed architecture is hierarchical in the smart city environment where a central broker (CB) collects/shares data from various IoT devices. All clients in the network connect to the central broker via the MQTT proxy, and the MQTT proxy establishes the connection with the CB on behalf of the clients.

The distributed brokers in the network have delegated the processing of a specific set of topics (e.g., s0/+/temp) managed by the central broker. Upon delegation, the subscription table of the corresponding topic is received as (1), as shown in Figure 4. The central management entity in the network notifies the MQTT proxy that a specific topic has been delegated, and the MQTT proxy then forwards the publication message for that topic to the distributed broker where the topic is delegated, as shown in (2). DB1 delivers a publication message of the topic to subscribers in the network. If there is a subscriber outside, then DB1 sends the message to the CB, as demonstrated in (3). The CB is responsible for delivering the message to external subscribers. Except for delegated topics, messages are passed directly to the CB without going through DB1, as illustrated in (4). Through this operation process, the proposed distributed broker architecture manages only a specific set of topics. Thus, the number of messages to be processed by the distributed broker can be reduced compared to the existing distributed MQTT architecture.

### 3.2. System Design

Based on considerations in the previous subsection, we propose a distributed MQTT architecture. The proposed architecture consists of distributed brokers and edge managers, distributed MQTT brokers, and MQTT proxies.

#### 3.2.1. Distributed Broker and Edge (DBE) Manager

**Figure 5:**
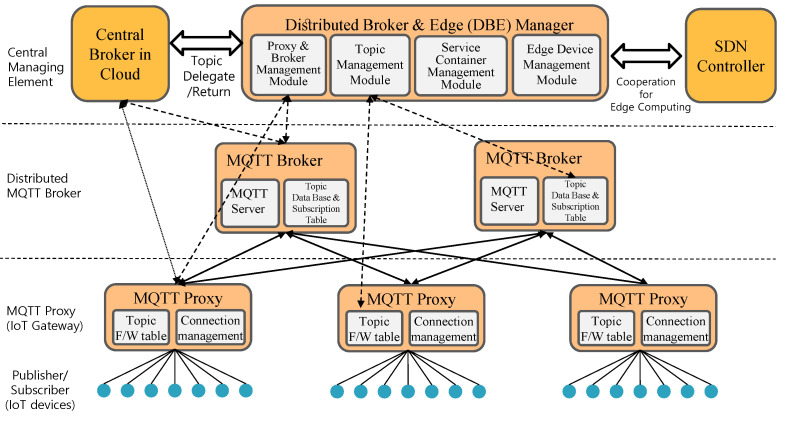
Distributed MQTT architecture based on edge computing.

First, the distributed broker and edge manager (hereinafter referred to as the DBE manager) centrally manages the edge computing resources within the network and consists of four management modules, as shown in Figure 5. Each management module performs the following functions.

- Proxy and Broker Management Module: This module manages MQTT proxy and broker containers on edge devices. This module manages the distributed MQTT broker and proxy containers and collects their status. It also registers and manages IoT gateways with MQTT proxy functions. If necessary, the proxy function can be operated as a container in the edge device. This management module can be delegated a topic or request a topic from a central broker. It also performs the function of returning the delegated topic.
- Topic Management Module: This module manages a table of delegated topics and a table of distributed brokers to which those topics are assigned. It notifies the MQTT proxy if it creates, redeploys, or deletes the distributed broker container so it can deliver the topic to the appropriate distributed broker container. It allows the MQTT proxy to deliver an MQTT message to the appropriate distributed broker when it receives a subscription/publication message from the IoT devices. The distributed broker container is relocated/deleted in the same way. In other words, this module takes charge of the topic, forwarding the table synchronization of the MQTT proxies.
- Service Container Management Module: This module manages the IoT application service container’s entry, placement, and deletion. It checks whether an application container uses specific topics generated by IoT devices on the local network. If the service container will be operated in an edge device using a specific topic generated by IoT devices in the network, then it requests a delegation to the central broker through the broker manager such that the topic can be provided locally through the distributed broker. Upon completion of the delegation request, the distributed broker and service container for the topic are placed together.
- Edge Device Management Module: This module collects and manages edge device status and statistics via the SDN controller. It manages edge devices’ computing resources (CPU, memory, and storage) on the local network. These data are used to decide whether or not to enter the service and broker container, as well as to optimize the placement.

**Figure 6:**
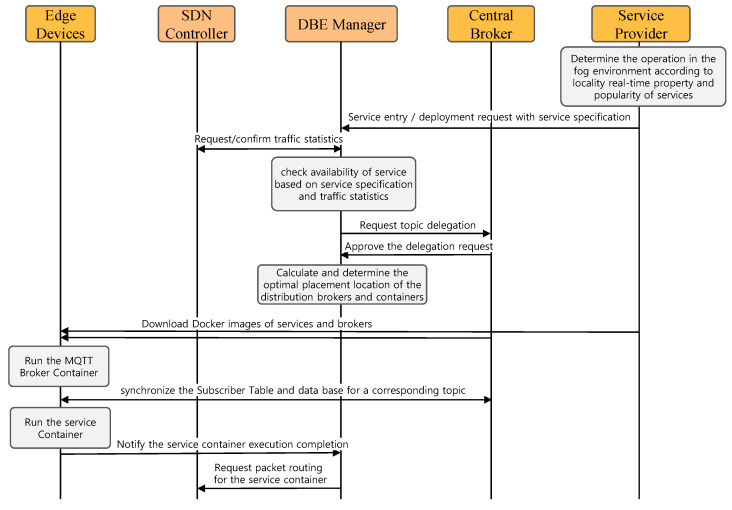
Edge-computing-based topic delegation and service entry process.

This DBE manager is placed in the local network. It collaborates with the SDN controller to monitor and control the network’s edge computing resources. The DBE manager tracks all container resource usage in the network and tracks each edge device’s available resources. Like the SDN controller, the DBE manager is a local object that manages edge resources. Figure 6 shows the edge entry process of a specific application service in the proposed architecture. First, the service provider evaluates the operational necessity in the edge environment locality, real-time property, and popularity of the service. If it is determined that the operation is necessary for the edge computing environment, then the edge computing environment is established and checked in the network, and service entry and placement are requested through the API of the DBE manager. The DBE manager sets statistical conditions at the SDN controller to obtain traffic statistics corresponding to the service for a certain period. It is to check the traffic statistics used by the service in the current network based on the specification of the service. The DBE manager then determines whether it can deploy based on the current edge computing’s service description, traffic statistics, and available resource information. The DBE manager asks the central broker to delegate the topic if the placement is possible. Upon approval of delegation from the central broker, the optimal placement location of the distributed broker and service container is calculated considering the traffic statistics and available edge computing resources.

After determining the container placement location, the edge device downloads the service and broker’s Docker images from their respective repositories. The edge device that finished downloading runs first from the MQTT broker container and waits for synchronization of the table of delegated topics between the distributed and central brokers. During this step, the DBE manager informs the MQTT proxy that the topic has been delegated to a distributed broker within the network. After that, the edge device executes the service container. When each container is placed in another edge device, it is controlled sequentially by the DBE manager. In the end, the DBE manager receives the service container execution completion message from the edge device and requests a packet routing rule modification from the SDN controller, if necessary, and ends the procedure for the service entry request.

#### 3.2.2. Distributed MQTT Broker and MQTT Proxy

In the proposed architecture, a distributed MQTT broker operates on a containerized edge device. Unlike a central broker, a distributed MQTT broker only manages specific topics under the control of the topic management module. It manages the subscriber table for delegated topics and sends the published message to subscribers. The distributed MQTT broker also has a topic database for storing the most recent data published for the topic with the Retain field. The broker sends the stored data to the new subscriber. When the distributed MQTT broker container starts and the DBE manager delegates the topic, the central broker synchronizes the subscription table and data. The MQTT proxy relays the client connection to support an environment where a distributed broker is dynamically created/moved, as in an edge computing environment. A similar concept is proposed in [19] and performs its functions at the gateway. The gateway connects all connected clients to a specific distributed broker based on the broker’s location and load. Unlike the gateway in [19], the MQTT proxy delivers the client message to a specific distributed broker according to the topic, and the messages for all other topics except for the delegated topics are transmitted to the central broker. The MQTT proxy is implemented in networking devices such as IoT gateways or Wi-Fi APs connected to edge devices and delivers the packets from/to all clients through a wired or wireless connection. A device such as a desktop computer connected to a network is fixed, and it is easy to install and distribute the program. Therefore, the installation program download function can be implemented in the DBE manager to let the wired device perform the proxy function by itself.

**Figure 7:**
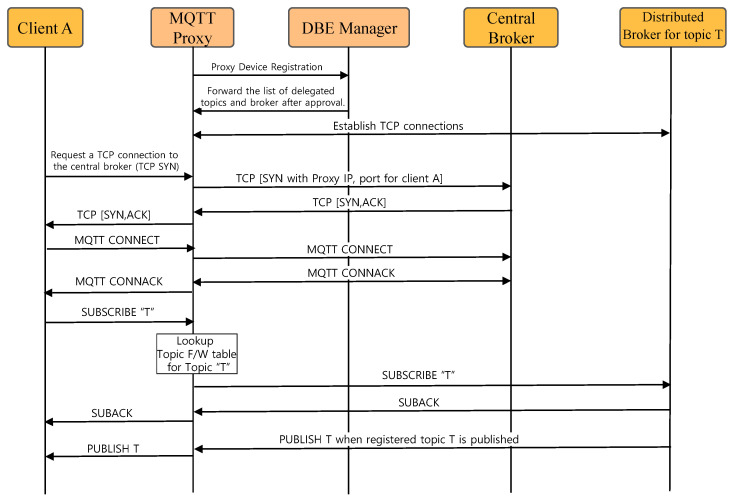
Operation of MQTT proxy-based client connection and subscription.

Figure 7 shows the operation procedure of the MQTT proxy-based client connection and subscription. First, when the proxy device initially connects to the network, it forwards the registration message to the DBE manager. The DBE manager sends the approval message and lists of delegated topics and distributed brokers. After that, the MQTT proxy establishes a TCP connection for client packet tunneling to the distributed brokers in the list. When the proxy receives a TCP SYN packet from client A, who wants to connect to the central broker, the proxy establishes a TCP connection with the central broker with a randomly generated port number (PORT_FOR_A) to identify client *A*. After the TCP connection to the central broker is established, the MQTT proxy establishes the TCP connection with client A. The proxy stores a table having a tuple of <A_IP, A_PORT, PORT_FOR_A> to identify the client. When receiving a subscription message of topic *T* from client *A*, the MQTT proxy checks the list of delegated topics and distributed brokers. If topic *T* is managed by a distributed broker, then the subscription message is transmitted to the corresponding broker in the form of [PORT_FOR_A, LENGTH_SUBSCRIBE_MSG, SUBSCRIBE_MSG] using the TCP connection established for tunneling. The distributed broker sends a reply for the subscription in the form of [PORT_FOR_A, LENGTH_SUBACK, SUBACK]. The proxy identifies the client through PORT_FOR_A and relays this message over the established TCP connection with the client. When a subscription-requested topic is issued, it is delivered in the same manner using tunneling.

## 4. Optimized Service Container Placement

As mentioned previously, the edge computing environment has limited computing resources, and thus, it is necessary to utilize the network resources efficiently. As the MQTT broker and IoT service are operated as a container in the edge devices in the network, the container placement decision impacts the operation efficiency.

### 4.1. Considerations for Optimizing Service Container Placement

First, the proposed architecture includes edge-enabled SDN switches managed by an SDN controller and a BE manager. Thus, when utilizing edge computing resources, not only computing but also networking resources should be considered. In this paper, the DBE manager optimizes container placement to reduce power consumption from computing resources usage and data transmission from service and distributed broker containers. Next, as many brokers and service containers as possible should be able to operate within the workload capacity of the network’s edge devices. In other words, the workload required for each container operation and the workload remaining for each edge device must be considered when determining container placement. It is similar to the 0–1 knapsack problem and is known as an NP-complete problem, given that the BE manager cannot arbitrarily divide specific service containers and operate them across multiple devices.

It is also important to consider the inter-dependencies between services and broker containers. For example, let us assume that there is a distributed broker *C* that manages a specific topic *T* and an IoT service container *S* that uses that topic *T*. In this case, placing two containers on the same edge device reduces network traffic power consumption. However, if two containers cannot be placed on the same device due to resource constraints, the effect of each container’s location must be considered. In such a complex environment, approximate solutions, such as the greedy algorithm, may be far from optimal. This leads to inefficient resource management at the edge. In this paper, the container placement optimization problem for reducing power consumption is formulated using ILP.

### 4.2. Problem Formulation

This sub-section describes variables, constraints, and objective functions for the optimization model based on the considerations mentioned above. The symbols used in this paper are described in Table 1.

#### 4.2.1. Optimization Variables and Constraints

The edge device set *D* includes all network devices with computing resources in the network and consists mainly of an edge-enabled SDN switch. The service set *S* includes all IoT services that operate using the edge computing resource in a container form. Topic set *T* includes all the topics delegated from the central broker. The distributed brokers that manage these topics are placed and operated in the edge device in a container form as in the case of the IoT service.

The distributed broker container that manages the delegated topic t∈T must be placed in one edge device *d*, which can be expressed as follows:

(1) $$\alpha_{\mathrm{td}}\in \left\{0,1\right\},\;\forall t\in T,d\in D$$

If the binary variable $\alpha_{\mathrm{td}}$ is 1, then the distribution broker container for topic *t* is placed in device *d*; otherwise, it is 0. As the distributed broker container for topic *t* must be placed in one edge device, it can be expressed as follows:

(2) $$\sum_{d\in D}\alpha_{\mathrm{td}}=1,\;\forall t\in T$$

Similarly, a container for a service *s* should be placed in the edge device *d*, which can be expressed as follows:

(3) $$\beta_{\mathrm{sd}}\in \left\{0,1\right\},\forall s\in S,d\in D$$

If a binary variable $\beta_{\mathrm{sd}}$ is 1, the container of the service *s* is placed in the device *d*; otherwise, it is 0. As in the case of a distributed broker, a container of a service *s* must be placed in one edge device, which can be expressed as:

(4) $$\sum_{d\in D}\beta_{\mathrm{sd}}=1,\;\forall s\in S$$

Thus, the distribution broker and service container placement are determined using the binary variables $\alpha_{\mathrm{td}}$ and $\beta_{\mathrm{sd}}$, respectively. The workload of the containers operating on each device cannot exceed the device’s free workload capacity. The workload used in this paper represents the average usage of computing resources such as the CPU, memory, and storage used by a specific container per unit time and is the normalized value used in a previous study [30]. The service container may have a different workload per input of traffic depending on service types. For example, if the service filters and stores input data only when certain conditions are satisfied, and it provides this information to the user, the workload per input data traffic is low. However, if a service is required for image processing, such as vehicle number identification, the workload per input traffic is significantly high. Therefore, the container workload per input traffic can be expressed as the constant value $\omega_{m}^{s}$ and $\omega_{u}^{s}$ according to the service. $\omega_{m}^{s}$ and $\omega_{u}^{s}$ indicate the workload according to the MQTT data of the service container and the workload according to the user’s non-MQTT data, respectively. However, in the case of the MQTT broker container, the workload per input data can be expressed as the constant $\omega_{m}^{b}$, as there is no significant difference according to the topic.

The distributed MQTT broker and service containers operating on each device generate the workload according to the input traffic, and these should not exceed the available workloads of the device. Thus, the following constraints are defined:

(5) $$w_{d}^{b}=\sum_{t\in T}\alpha_{td}\omega_{b}^{m}\left(\sum_{d\in D}\tau_{td}+\sum_{s\in S}\delta_{sd}\right)$$

(6) $$w_{d}^{s}=\sum_{s\in S}\beta_{sd}\left(\omega_{m}^{s}\sum_{t\in T}\delta_{sd}+\omega_{u}^{s}\sum_{d\in D}\delta_{sd}\right)$$

(7) $$w_{d}^{b}+w_{d}^{s}\leq C_{d},\;\forall d\in D$$

where $\tau_{td}$ represents the average traffic bandwidth for the topic *t* generated by the users and IoT devices connected to the edge device *d*, which is an SDN switch. $\delta_{sd}$ represents the average traffic bandwidth for the service *s* generated by the users and the IoT devices connected to the edge device *d*. Thus, Equation (7) indicates that the workload sum $e_{d}^{b}$ of the distributed broker containers and the workload sum $e_{d}^{s}$ of the service containers operated in a device *d* should not exceed *d*’s workload capacity $C_{d}$.

#### 4.2.2. Objective Function

The BE manager in the proposed architecture sets the containers to reduce the power consumption due to edge computing in the local network while considering the workload of the containers and workload capacity of the devices. First, the power consumption due to the container operating workload in each device $e_{d}$ is defined as follows:

(8) $$e_{d}=\varepsilon_{d}\left(w_{d}^{b}+w_{d}^{s}\right)$$

where $\varepsilon_{d}$ represents the power efficiency per workload of the device *d* and may have different values depending on the performance of each edge device. In addition, the total amount of network traffic that should be forwarded according to the container placement in the network varies, and the power consumption due to this should be calculated. The total traffic $\mu_{Total}$ in the network due to edge computing can be defined as follows:

(9) $$\mu_{\mathrm{Total}\;}=\mu_{bd}+\mu_{sd}+\mu_{bs}$$

where $\mu_{bd}$ is the total traffic of the MQTT data between the distributed broker container *b* and the IoT and user devices connected to the edge device *d*. $\mu_{sd}$ represents the total traffic of the non-MQTT data generated between the service container *s* and the IoT and user devices connected to the edge device *d*. $\mu_{bs}$ represents the total traffic of the MQTT data between the distributed brokers and service containers. The components of Equation (9) are defined as follows:

(10) $$\mu_{bd}=\sum_{t\in T}\sum_{d\in D}\sum_{d^{\prime }\in D}\alpha_{{\mathrm{td}}^{\prime }}h_{{\mathrm{dd}}^{\prime }}\tau_{\mathrm{td}}$$

(11) $$\mu_{sd}=\sum_{s\in S}\sum_{d\in D}\sum_{d^{\prime }\in D}\beta_{sd}\delta_{s{\mathrm{sd}}^{\prime }}h_{{\mathrm{dd}}^{\prime }}$$

(12) $$\mu_{bs}=\sum_{t\in T}\sum_{d\in D}\sum_{d^{\prime }\in D}\sum_{s\in S}\alpha_{\mathrm{td}}h_{{\mathrm{dd}}^{\prime }}\beta_{{\mathrm{sd}}^{\prime }}\delta_{\mathrm{st}}$$

Based on the above equations, the power consumption $e_{N}$ due to the total network traffic caused by edge computing can be expressed as follows:

(13) $$e_{N}=\varepsilon_{N}\times \mu_{\mathrm{Total}\;}$$

where $\varepsilon_{N}$ is a constant value representing the power efficiency per network traffic, and the power consumption due to the network traffic can be calculated by multiplying the total traffic in the network by this constant. The objective function of the container placement for minimizing the power consumption in the network due to edge computing, which is expressed in Equations (8)–(13), can be expressed as follows:

(14) $$f\left(\alpha_{td},\beta_{sd}\right)=\sum_{d\in D}e_{d}+e_{N}$$

Equation (14) represents the sum of the power consumption due to edge computing according to the binary variables $\alpha_{td}$ and $\beta_{sd}$. The optimization problem is mathematically formulated as follows:

(15) $$\begin{array}{c} \min f\left(\alpha_{td},\beta_{sd}\right)\\ s.t.\;(1),\;(2),(3),(4),(7)\\ \forall t\in T,d\in D,s\in S \end{array}$$

The above optimization problem is a nonlinear integer programming problem owing to Equation (12). However, as Equation (12) consists of the product of the binary variables, we can reduce the computational complexity using the auxiliary variable. In order to linearize Equation (12), the auxiliary variable for the quadratic term of the binary variables is defined as follows:

(16) $$\psi_{{\mathrm{sd}}^{\prime }}^{\mathrm{td}}=\alpha_{\mathrm{td}}\beta_{{\mathrm{sd}}^{\prime }}\forall t\in T,s\in S,\;d,d^{\prime }\in D$$

This auxiliary variable $\psi_{sd^{\prime }}^{td}$ has the following constraints:

(17) $$\psi_{{\mathrm{sd}}^{\prime }}^{\mathrm{td}}\in \left\{0,1\right\},\;\forall t\in T,s\in S,\;d,d^{\prime }\in D$$

(18) $$\psi_{{\mathrm{sd}}^{\prime }}^{\mathrm{td}}\leq \alpha_{\mathrm{td}}$$

(19) $$\psi_{{\mathrm{sd}}^{\prime }}^{\mathrm{td}}\leq \beta_{{\mathrm{sd}}^{\prime }}$$

(20) $$\psi_{{\mathrm{sd}}^{\prime }}^{\mathrm{td}}\geq \alpha_{\mathrm{td}}+\beta_{{\mathrm{sd}}^{\prime }}-1$$

Using the auxiliary variable $\psi_{sd^{\prime }}^{td}$, Equation (12) is redefined as follows:

(21) $$\mu_{bs}=\sum_{t\in T}\sum_{d\in D}\sum_{d^{\prime }\in D}\sum_{s\in S}\psi_{\mathrm{sd}}^{\mathrm{td}}h_{\mathrm{dd}}\delta_{\mathrm{st}}$$

The optimization problem in Equation (15) can be redefined as an ILP problem as follows:

(22) $$\begin{array}{c} \min f\left(\alpha_{td},\beta_{sd},\psi_{s\;d}^{\mathrm{td}}\right)\\ s.t.\;(1),\;(2),(3),(4),(7),(17)-(20)\\ \forall t\in T,d,d^{\prime }\in D,s\in S \end{array}$$

## 5. Performance Evaluation

### 5.1. Evaluation of Distributed MQTT Broker Architecture

First, we performed simulations to compare the proposed distributed MQTT architecture to EMMA [19] and the SF method, which was described in Section 3. EMMA was chosen because it proposed a distributed MQTT broker that was dynamically allocated. EMMA moves clients to adjacent distributed brokers using gateways and allowing for dynamic creation and destruction of distributed MQTT brokers. When issuing/subscribing to a topic, EMMA behaves like SF. Unlike SF, EMMA does not flood externally for local topics because it assumes that local and global topics are separated beforehand. Consequently, the two methods differ in the size of the distributed broker’s subscription table. Although this is different from the proposed environment, which does not distinguish between global and local topics in advance, we performed simulations assuming that the topics that are not subscribed to by clients in external networks are local topics. Table 2 lists the simulation parameters. We modeled the behavior of subscribers/publishers and brokers in each architecture using MATLAB 2017a. Our experiment simulates IoT properties in a smart city environment, with a large number of devices, large amounts of data sharing, easy creation and removal of services, and multiple network owners. Thus, we test on a large number of devices in a multiple network set with 10,000 publishers and subscribers with 200 subscribed topics each. The topic name was 40 bytes long. The topic set the length for storing subscriber information at 6 bytes (4 bytes for IPv4 address and 2 bytes for TCP port). In a real environment, a larger memory and storage space is required for maintaining a subscription table, such as a hash table, subscriber information table, and topic tree information. In particular, a database for a topic with a Retain field set requires a lot of storage space. The size of the subscription table was calculated using the minimum amount of data that must be kept in memory. The policy determines which topic a distributed MQTT broker delegated. In this paper, policies are divided into three categories: Proposed LP, Proposed LP NS, and Proposed LP NS EP. Proposed LP represents a policy that delegates only those topics that have a pair of subscribers and publishers within the local network. Proposed LP NS represents a policy that delegates not only topics delegated by Proposed LP but also delegates topics that are currently published in the internal network but do not have subscribers. Proposed LP NS EP represents a policy that additionally delegates topics that are published from external networks but have two or more subscribers in the local network. Hereinafter, Proposed LP, Proposed LP NS, and Proposed LP NS EP are denoted by LP, NS, and EP, respectively.

**Figure 8:**
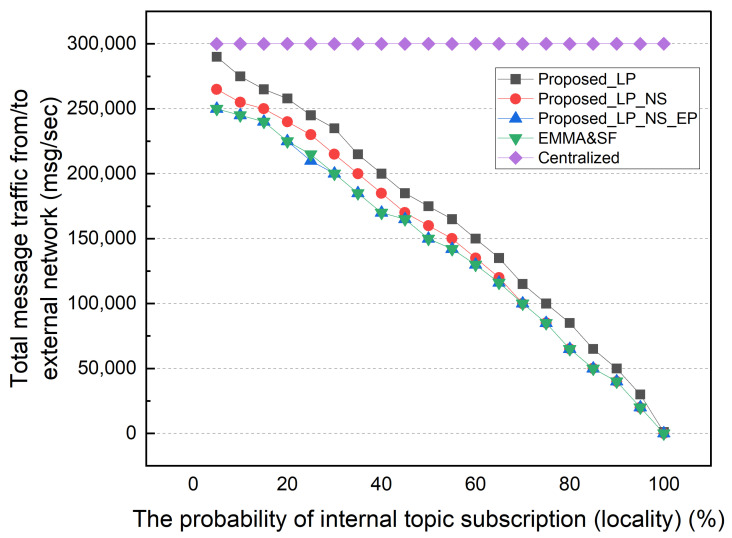
Message traffic between external networks by subscription locality.

First, we performed a simulation based on the subscription’s locality. In this simulation, the locality of the subscription is the probability of subscribing to a topic published on the local network where the subscriber is located. Figure 8 shows the total network message traffic between external networks by subscription locality. In this simulation, subscribers choose whether to subscribe to internal or external topics, and the topics are chosen at random from the uniform distribution. The simulation default setting for a central-broker-only architecture, which is denoted by “centralized” in the legend, is 10,000 publishers per network, one topic per second, and two subscribers per topic, resulting in 300,000 external network traffic items. In an environment with a very low locality of subscription at 5%, the LP scheme shows the highest external traffic, similar to that of the CB architecture. The NS scheme generates the next highest amount of external traffic. EP delegated all topics reducing external traffic. Therefore, it shows the same external traffic as EMMA and SF. As stated previously, the EMMA and SF methods generate identical external traffic except for subscriber table maintenance overhead and subscription message forwarding.

**Figure 9:**
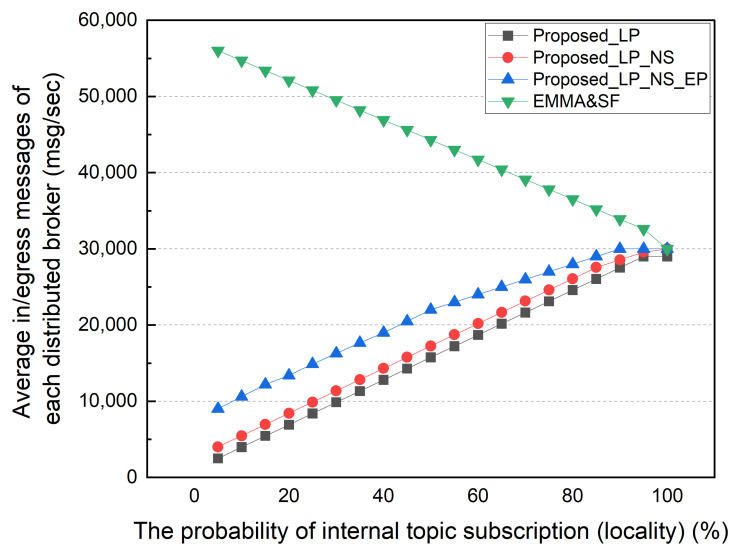
Average number of in/egress messages by subscription locality.

The amount of external traffic decreases as the subscription locality increases. The external traffic of all schemes is zero except for LP in a 100% locality of the subscription environment. Throughout LP, unsubscribed topics are sent directly to the CB rather than processed by the distributed broker, resulting in additional external traffic. An environment with 100% locality of the subscription is not realistic. As there is no need for distributed broker collaboration, an MQTT broker that operates independently for each network can be used. However, the existence of subscribers and services using various data published by different sources, such as a smart city environment, requires distributed broker collaboration. Figure 9 shows the average number of ingress/egress messages per distributed broker by locality. This result confirms the advantage of the distributed MQTT architecture proposed in this paper. Due to the fact that LP only delegates topics to internal subscribers, the average number of ingress/egress messages is significantly lower. NS and EP show significantly less MQTT messages processed by a distributed broker than EMMA and SF.

The proposed architecture aims to avoid wasting edge computing resources on distributed MQTT brokers. From this perspective, the proposed architecture is much more efficient than the SF and EMMA schemes in terms of computing resource usage. When the locality is low, the number of I/O messages per distributed broker is high. Message input/output occurs once for each distributed broker to which the publisher and subscriber are connected. The number of messages processed by the distributed broker in the proposed architecture, EMMA, and SF structures is similar as locality increases. Then, the distributed broker will handle most of the topics in the proposed architecture. In this simulation, we assume the proposed architecture delegated all topics matched to each policy without considering edge computing resource usage. However, when edge computing resources are limited, only topics used by an edge computing service or frequently used in the local network can be delegated.

**Figure 10:**
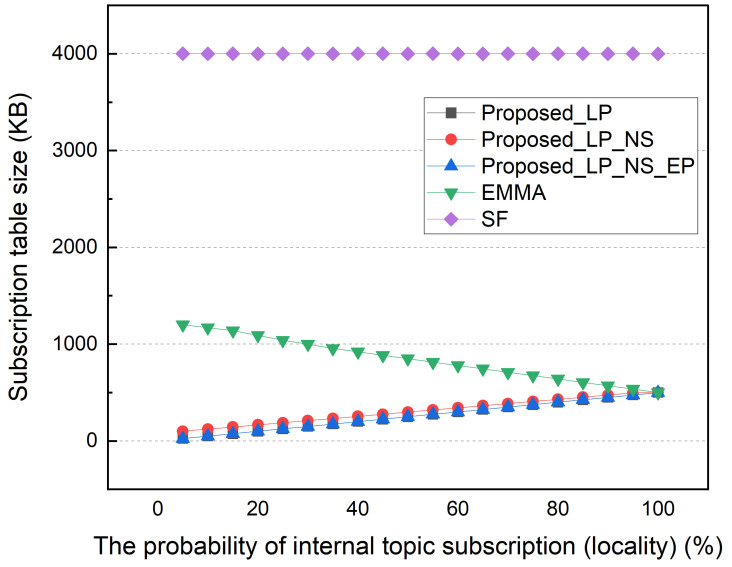
Subscription table size according to subscription locality.

As shown in Figure 10, each distributed broker’s average subscription table size varies by locality. The delegated topics subscription table represents the synchronization overhead between distributed and central brokers. As the SF scheme maintains the subscription table for all subscriber subscription messages, it requires a much larger subscription table than other schemes, regardless of locality. When separating the global and local topics, EMMA’s subscription table is smaller than the proposed scheme’s, but when the locality is low, it is much larger. As previously stated, we only considered the distributed broker’s minimum subscription table size. A hash table for improving the retrieval speed and storage space for maintaining the data of the topic in which the retained field is set were not reflected. Since EMMA supports the dynamic creation/destruction of distributed brokers, it is necessary to synchronize these data, resulting in a larger synchronization overhead than the subscription table. Because the SF and EMMA methods produce identical results, we compare the proposed methods to only the EMMA method in the simulation.

**Figure 11:**
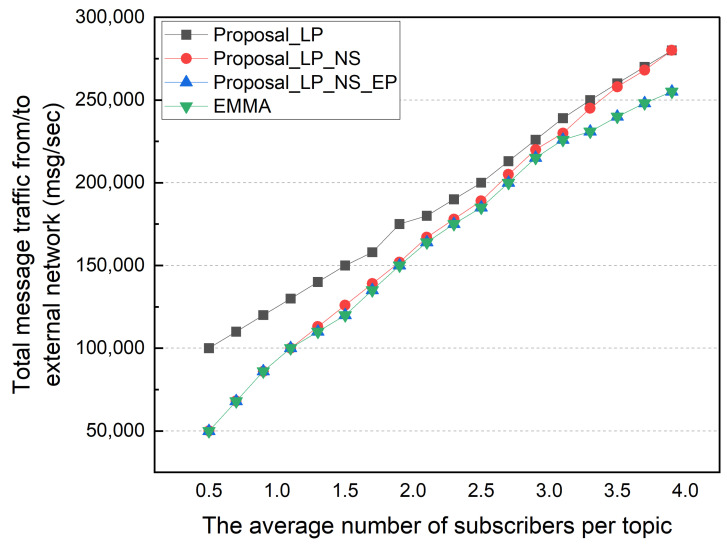
Message traffic between external networks by subscribers per topic.

**Figure 12:**
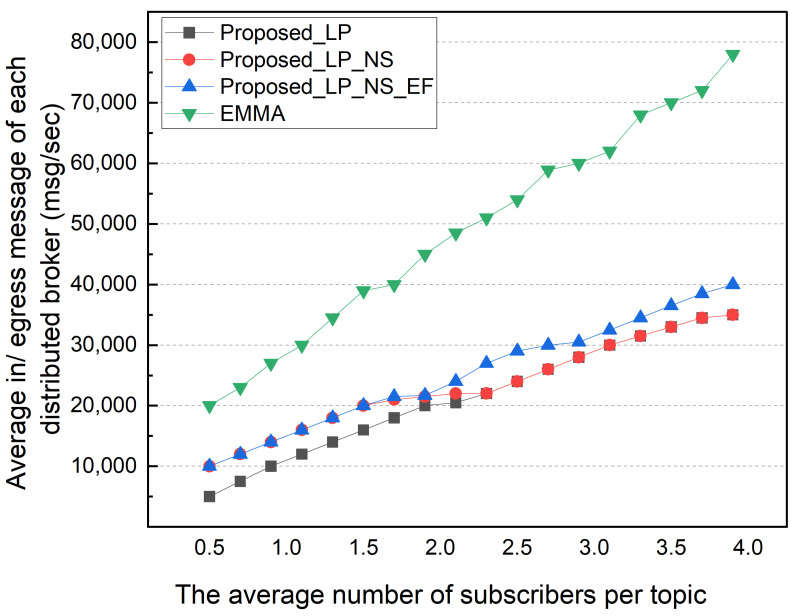
Average of ingress/egress messages according to subscribers per topic.

Afterward, we performed a simulation based on the average number of subscribers per topic. The number of subscription topics per subscriber was changed from 50 to 400, so the average number of subscribers per topic varied from 0.5 to 4.0. The locality was fixed to 50% in this simulation. Figure 11 shows the total external message traffic according to the average number of subscribers per topic. As the number of subscribers increases, the total external message traffic gradually increases. The EMMA and EP methods show the same total external message traffic. In an environment with an average of 0.5 subscribers, the LP method has twice the external traffic as the other methods. This is because of a high portion of topics with no subscribers in an environment with a low average number of subscribers per topic. The NS, EP, and EMMA schemes with no subscribers show low external traffic. As the average number of subscribers per topic increases, this difference is somewhat reduced; and in an environment with 3.9 average subscribers per topic, the total external traffic in the NS and LP schemes is nearly identical. The difference between the NS and EP methods increases as the average number of subscribers per topic increases. This is due to an increase in the number of topics with multiple subscribers in the local network that are published externally. Figure 12 shows the average ingress/egress messages per distributed broker based on the number of subscribers per topic in the same simulation environment. Similar to previous results, the number of messages processed in each distributed broker increases as the average number of subscribers per topic increases. When compared to EMMA, the EP method has a lower average number of messages processed in each distributed broker, despite having the same total external traffic results.

**Figure 13:**
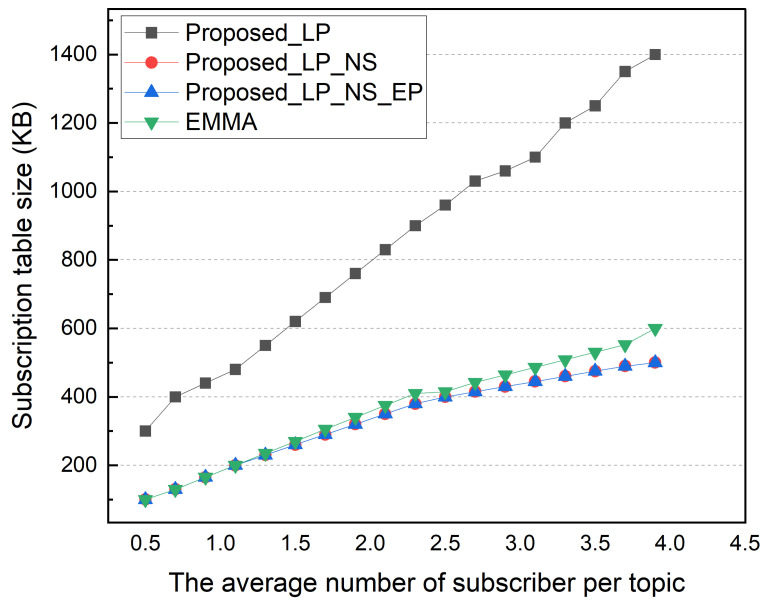
Subscription table size according to number of subscribers per topic.

In addition, as the average number of subscribers per topic increases, it can be seen that the EMMA increases with a higher slope than the proposed method. Figure 13 shows the size of the subscription table based on the average number of subscribers per topic. A pattern resembling previous simulation results is seen. The NS and LP schemes share the same subscription table size because topics without subscribers are not maintained. Although the size of the storage for topic data is not evaluated in this paper, there is a difference in space for storing topic data when the retained field is set. Similar to previous simulation results, EMMA’s subscription table size increases with a higher slope than the proposed architecture as the average number of subscribers per topic increases.

Finally, we performed simulations based on the topic’s popularity distribution. In this simulation, the subscriber selects a topic based on the Zipf distribution probability ($rank^{-K}/\sum rank^{-K}$) rather than selecting the topic with a uniform distribution probability. The popularity concentration represented as K defines the intensity of the popular topic to be selected. The value of K varied from 0.1 to 2. Topics with a higher popularity rank are selected more intensively as K increases. If K is very small, it becomes close to a uniform distribution. In this simulation, the subscription locality was fixed to 50%.

**Figure 14:**
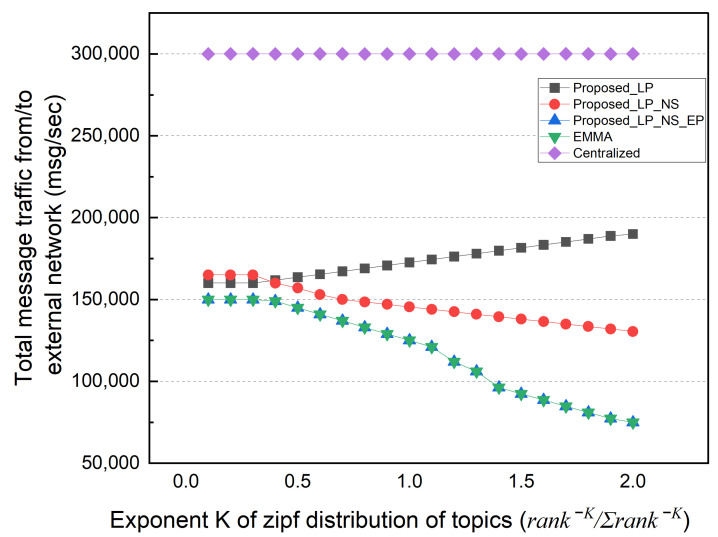
Message traffic between external networks according to topic popularity.

**Figure 15:**
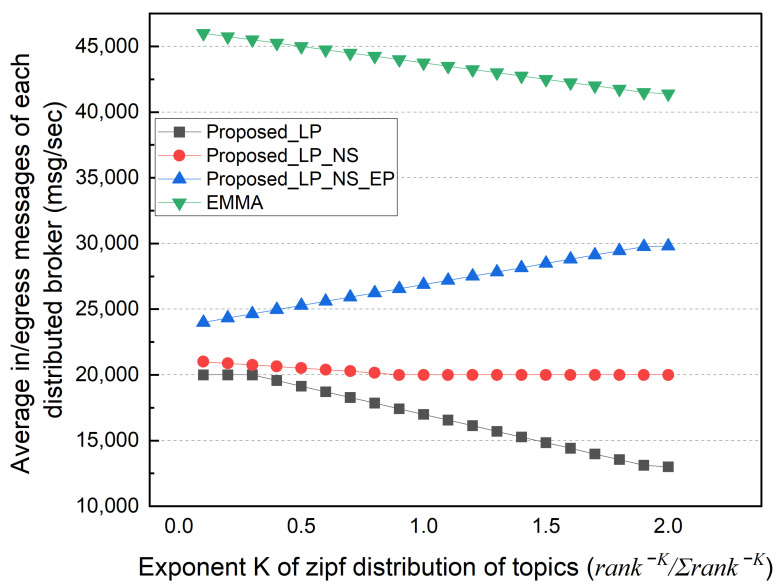
Average number of in/egress messages according to topic popularity.

**Figure 16:**
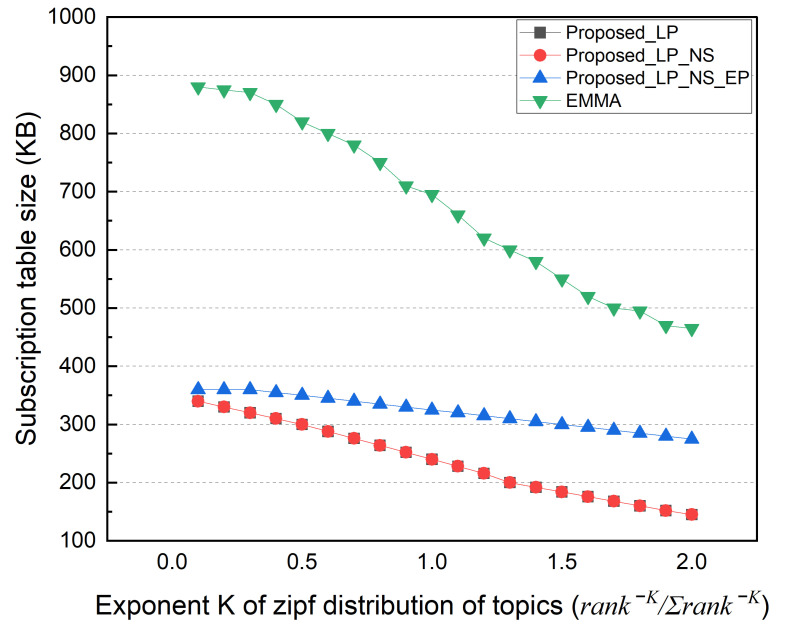
Subscription table size according to popularity concentration of topics.

Figure 14 shows the total external message traffic as K changes. The EMMA and EP schemes have the same total external traffic, which decreases as the popularity concentration of each topic increases. Even if the topic is published by an external network, the EP scheme can deliver it to multiple subscribers in the local network with only one reception. In contrast, as the K value rises, the number of topics with no subscribers increases. Therefore, the total external traffic of the LP scheme increases, but the total external traffic of the NS scheme decreases. Figure 15 shows the distributed broker’s average number of ingress/egress messages. In the EP method, the distributed broker’s average I/O messages increase as the external traffic decreases. Similarly, in the LP method, the number of messages that a distributed broker has to process decreases as much as the number of external traffic rises. Because EMMA can deliver the topic to multiple subscribers in the local network with only one reception, the average number of ingress/egress messages decreases with increasing popularity concentration. Figure 16 shows the size of the subscription table in the same simulation; the table size decreases as K increases. As the number of unsubscribed topics increases, the number of topic lists to keep in the subscription table decreases. Furthermore, the number of subscribers to the same topic grows. The topic name is longer than the subscriber information in the simulation, increasing the subscription table size.

In summary, the proposed distributed broker architecture can choose the LP, NS, and EP delegation policy based on the distributed broker’s purpose. The EP method generates fewer ingress and egress messages than the existing distributed MQTT broker architecture while generating the same amount of external traffic. This can reduce the distributed broker’s message processing latency. The LP and NS methods generate more external traffic than EMMA, but they reduce the number of messages that a distributed broker must process. This reduces the use of edge resources by the distributed broker. It also reduces synchronization overhead in environments where the distributed MQTT broker can be deployed dynamically by requiring smaller subscription table sizes.

### 5.2. Evaluation of Service Container Placement Optimization

In Section 4, we designed an IoT service and distributed MQTT broker container placement optimization to reduce power usage. This subsection examines the simulation results to assess the proposed container placement scheme. The simulation parameters are shown in Table 3. Some parameters, such as the power efficiency of the workload and network traffic, were set based on existing research [24,30]. The service workload includes tasks such as computing and data downloading for navigation services in smart cities. The user must download data and maps while calculating the best route. We assumed a partially connected mesh topology with 10 Gbps links between edge devices. The simulations were run in an edge computing environment with many IoT and user devices connected to the edge device generating MQTT and IoT service traffic.

Our proposed scheme is compared to two other schemes. (i) The greedy method sorts distributed MQTT brokers and service containers in order of required workload, then allocates them sequentially with the lowest power consumption while considering the edge device’s remaining workload capacity. (ii) The random method arbitrarily allocates brokers and service containers based on the remaining workload capacity. The simulation parameters, such as device workload capacity and power efficiency, have arbitrary values. The result is a mean of at least 20 simulations.

**Figure 17:**
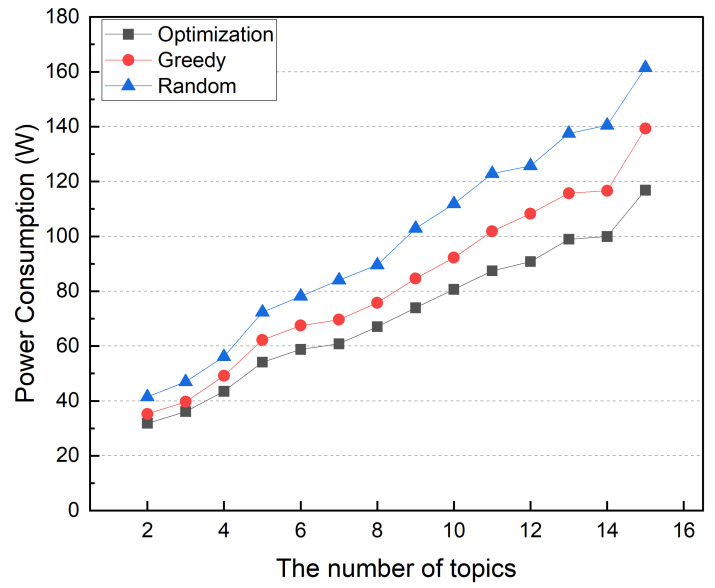
Power consumption according to the number of topics.

First, we ran a simulation to see how the number of topics managed in edge computing affected the results. Figure 17 shows the power consumption of a distributed broker based on the number of delegated topics. The number of edge devices and services is fixed at 6, but the number of topics is variable. Each service uses an average of 20% of the delegated topics. The simulation shows that as the number of topics increases, the power consumption of all container placement schemes increases. It is because the MQTT data traffic increases as the number of topics increases.

**Figure 18:**
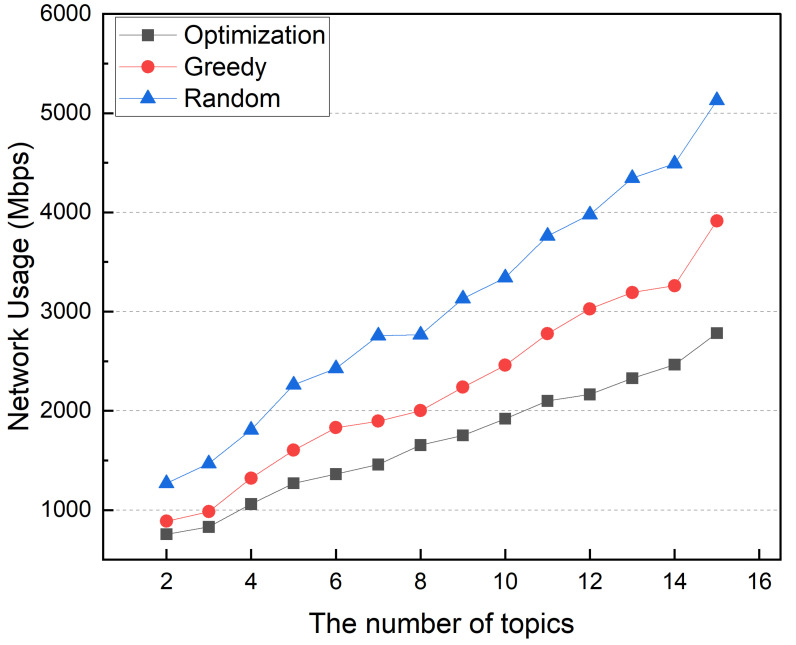
Network usage according to the number of topics.

The workload of distributed MQTT brokers and IoT service containers depends on input traffic. Thus, computing resource usage increases power consumption. When the number of topics is small, the optimization and greedy methods are similar. When the number of topics is small, the network traffic between the MQTT broker and the IoT service container is small. However, as the number of topics grows, the power consumption difference between greedy and optimization methods grows. An increased number of topics increases the interdependency of MQTT brokers and service containers. The greedy method uses more power than the optimization method because it places containers without considering interdependencies between MQTT broker and IoT service containers. Figure 18 shows the average network usage in this simulation, which follows the same trend as the power consumption. Unlike the greedy method, the optimization scheme determines container placement while considering interdependency between distributed broker and IoT service containers, resulting in lower power consumption and reduced network traffic.

**Figure 19:**
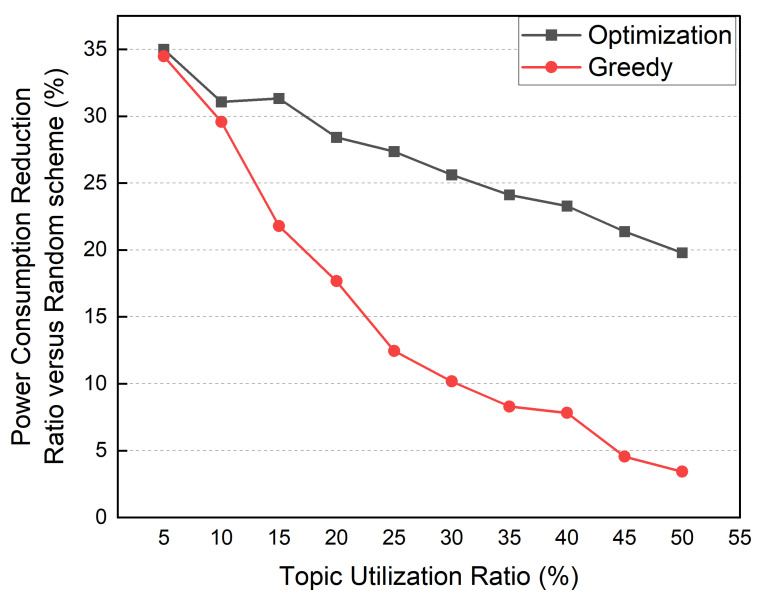
Power consumption comparison according to the topic utilization ratio.

Next, we performed a simulation to evaluate the effect of the interdependency between the distributed MQTT broker and service container. The number of edge devices and services was fixed at 6, and the number of topics was fixed at 10. The average topic utilization ratio ranged from 5% to 50%. Figure 19 compares the power reduction ratio (PCRR) to the random method. Since the service container is affected by the location of multiple distributed broker containers, the PCRR of the optimization and greedy methods decreases. As the interdependence between the service and the broker container is low, the greedy and optimization methods show similar PCRRs in environments where the service’s average topic usage rate is 5–10%. The greedy method’s PCRR decreases sharply as the average topic utilization ratio rises. A PCRR of less than 5% is obtained in environments with an average topic utilization ratio of 50%. In contrast, the optimization method shows a PCRR of 20% even in an environment with an average topic utilization ratio of 50%.

**Figure 20:**
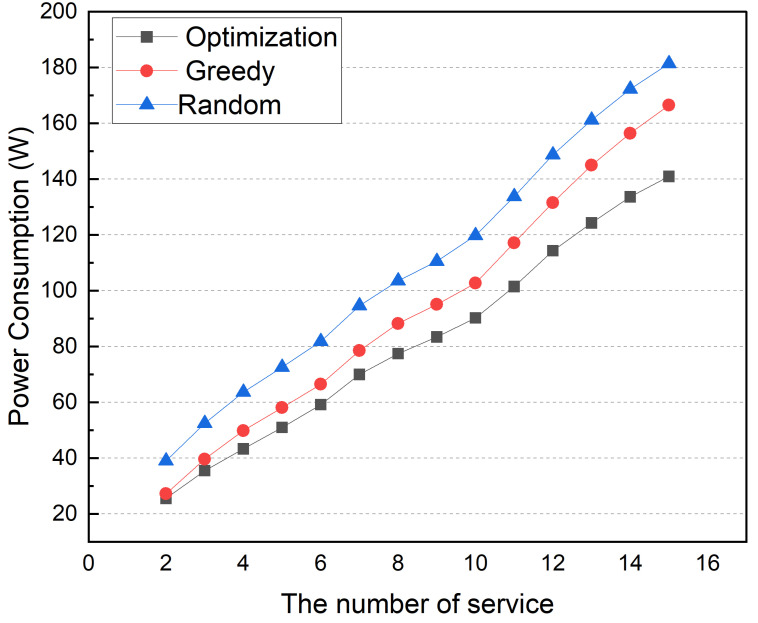
Power consumption according to the number of services.

Figure 20 shows the simulation results according to the number of services. In this simulation, the number of edge devices and topics is fixed at 6, and the average topic utilization ratio is fixed at 20%. The number of services ranged from 2 to 15. As shown in Figure 20, the power consumption of all placement methods increases with the number of services. Since the service container generates more workload than the broker container, the overall power consumption is higher than in Figure 17. The greedy and optimization methods consume power in a similar way to the previous simulation on a number of topics. The simulation results show that the power consumption difference between the greedy and optimization methods grows as the number of services grows.

**Figure 21:**
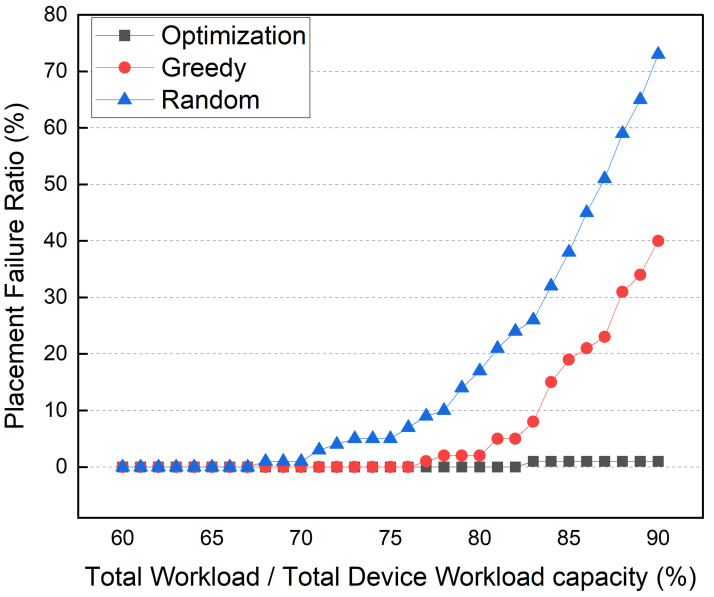
Container Placement Failure Ratio.

Then, we ran a simulation to see how the workload capacity of the edge devices affected container placement. Furthermore, the container placement method should be able to operate as many broker and service containers as possible without overloading the edge devices in the network. From this perspective, the optimization method may be superior to the greedy method. Figure 21 shows the container placement failure ratio according to the total edge device workload capacity. As shown in the graph, the x-axis represents the required workload for all broker and service container operations compared to the total workload capacity of all edge devices (WR). In this simulation, there were six edge devices, ten topics, and services. After the other simulation parameters were set to arbitrary values, the workload capacity of each of the devices was adjusted to a specific WR value. The simulation is performed 100 times for each WR. The container placement failure ratio was calculated by counting the cases wherein each method failed to place all the containers due to the edge device workload capacity. The random method fails to place containers at 70% WR in the simulation. The container placement failure ratio rapidly increases at 80% and reaches 70% when the WR is 90%. Since the greedy method places the container with the most workload first, it outperforms the random method. When the WR is 80%, the greedy method’s container placement failure rate increases. The greedy method also has a 40% placement failure rate at 90% WR. However, even at 90% WR, the optimization method shows low container placement failure rates. Despite its higher computational complexity and longer search time, these simulation results demonstrate the need for optimization-based container placement.

**Figure 22:**
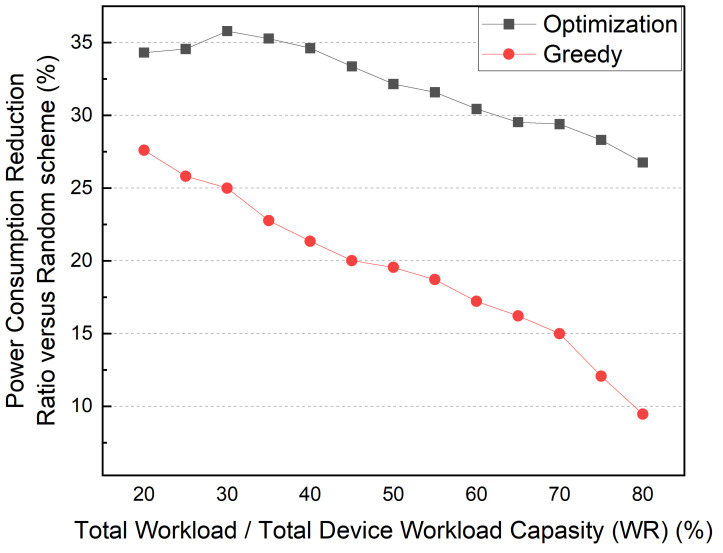
Power consumption comparison according to WR.

Finally, Figure 22 shows the PCRR according to the WR. The number of devices and services was fixed at six, and the number of topics was fixed at ten. The WR ranged from 20% to 80%. For a fair comparison, we averaged the simulation results performed in the environment wherein container placement failures were not observed in the case of all the methods. As shown in Figure 22, the greedy method reduces PCRR as WR increases. In particular, when the WR is 80%, the PCRR is decreased to 10%. When the WR is 40% or less, the optimization method shows a constant PCRR. However, even when the WR is 80%, the optimization method shows a 26% PCRR.

In summary, as the network grows in topics and services, edge computing increases overall power consumption. In most cases, the proposed method uses less power than other methods. In particular, in the environment with a high WR, the proposed method shows a lower deployment failure ratio and higher power consumption reduction ratio compared with the other schemes.

## 6. Conclusions

Developing efficient edge computing architecture for a smart city IoT environment is necessary to obtain low latency and energy resource consumption. This paper proposed a distributed broker architecture that adapts the MQTT of the publisher/subscriber structure with edge computing technology. Thus, the edge layer’s application service does not need to directly connect with a remote cloud server. In the proposed architecture, each distributed broker has delegated the management of a specific topic under the control of the DBE manager. Edge resource usage and broker operation can be reduced by managing only the designated topic. We also formulated an ILP problem for optimizing service container placement over distributed edge computing resources and compared its performance to other simulation-based techniques. The proposed placement method could efficiently distribute the subscribed topic’s service instance to the distributed edge resources. We compare our proposed architecture to EMMA, an existing distributed MQTT middleware. Our architecture reduces the number of ingress/egress messages while generating the same number of external traffic messages as the existing architecture. Reducing the average number of messages per distributed broker also reduces processing latency and the need for edge resources. Because our scheme requires smaller subscription tables, it reduces synchronization overhead. We also evaluate our container placement optimization scheme using distributed MQTT brokers and service containers, then results in our method using fewer network resources and less power.

In the future, we will implement a distributed broker and evaluate the proposed architecture’s performance in terms of latency, required memory, and storage in a real environment.

## Figures and Tables

**Table 1:** Notations used in proposed optimization model.

Symbol	Description
*S*	Set of IoT services in the network
*D*	Set of edge devices (SDN switch with edge computing resource)
*T*	Set of delegated topics
$C_{d}$	Workload capacity of edge device *d*
$\alpha_{\mathrm{td}}$	A binary variable that indicates whether a container *t*
	of a distribute MQTT broker of topic is on a device *d* or not
$\beta_{\mathrm{sd}}$	A binary variable that indicates whether a
	container of a service *s* is in a device *d*
$\tau_{\mathrm{td}}$	Average traffic bandwidth generated by
	clients connected to device *d* for topic *t*
$\delta_{\mathrm{sd}}$	Average traffic bandwidth generated by
	clients connected to device *d* for service *s*
$\omega_{m}^{b}$	Workload per traffic of the distributed
$\omega_{m}^{s}$	Workload per traffic of the distributed
	MQTT broker container
$\omega_{u}^{s}$	Workload per MQTT traffic of the
	application container of service *s*
$h_{ij}$	Hop count between edge devices *i* and *j*
$\varepsilon_{d}$	Device *d*’s power efficiency per workload
$\varepsilon_{N}$	Power efficiency per network traffic bandwidth

**Table 2:** Simulation parameters for distributed MQTT architecture.

Description	Default Value	Unit
Number of networks	10	Networks
Number of publishers per network	10,000	Publishers
Publishing rate per publisher	1	Msg/s
Number of subscribers per network	100	Subscribers
Number of subscribed topics per subscriber	200	Topics
Length of topic name	40	Byte
Length of subscriber information (IP, port)	6	Byte

**Table 3:** Simulation parameters for container placement optimization.

Symbol	Description	Value	Unit
$C_{d}$	Workload capacity of the edge device *d*	20,000–40,000	Workload
$\omega_{m}^{b}$	Distributed broker container workload per	10	Workload/Mbps
	MQTT traffic The workload per MQTT		
$\omega_{m}^{s},\omega_{u}^{s}$	and user traffic of the application container of service *s*	1–50	Workload/Mbps
$\varepsilon_{d}$	Device *d* ’s power efficiency per workload	1.3–2.5 × $10^{-1}$	mW/Workload
$\varepsilon_{N}$	Power efficiency network traffic	$1.37\times 10^{-2}$	mW/Mbps
